# Identification of Antimicrobial Resistance-Associated Genes through Whole Genome Sequencing of *Mycoplasma bovis* Isolates with Different Antimicrobial Resistances

**DOI:** 10.3390/pathogens9070588

**Published:** 2020-07-19

**Authors:** Lisa Ledger, Jason Eidt, Hugh Y. Cai

**Affiliations:** Animal Health Lab, University of Guelph, 419 Gordon St., Guelph, ON N1H 6R8, Canada; lledger@uoguelph.ca (L.L.); jeidt@uoguelph.ca (J.E.)

**Keywords:** *Mycoplasma bovis*, antimicrobial resistance, whole genome sequencing, MIC, cgMLST

## Abstract

Antimicrobial resistance (AMR) in *Mycoplasma bovis* has been previously associated with topoisomerase and ribosomal gene mutations rather than specific resistance-conferring genes. Using whole genome sequencing (WGS) to identify potential new AMR mechanisms for *M. bovis,* it was found that a 2019 clinical isolate with high MIC (2019-043682) for fluoroquinolones, macrolides, lincosamides, pleuromutilins and tetracyclines had a new core genome multilocus sequencing (cgMLST) type (ST10-like) and 91% sequence similarity to the published genome of *M. bovis* PG45. Closely related to PG45, a 1982 isolate (1982-M6152) shared the same cgMLST type (ST17), 97.2% sequence similarity and low MIC results. Known and potential AMR- associated genetic events were identified through multiple sequence alignment of the three genomes. Isolate 2019-043682 had 507 genes with non-synonymous mutations (NSMs) and 67 genes disrupted. Isolate 1982-M6152 had 81 NSMs and 20 disruptions. Using functional roles and known mechanisms of antimicrobials, a 55 gene subset was assessed for AMR potential. Seventeen were previously identified from other bacteria as sites of AMR mutation, 38 shared similar functions to them, and 11 contained gene-disrupting mutations. This study indicated that *M. bovis* may obtain high AMR characteristics by mutating or disrupting other functional genes, in addition to topoisomerases and ribosomal genes.

## 1. Introduction

*Mycoplasma bovis* is a member of the Mollicutes; membrane-bound bacteria which lack a cell wall, precluding the use of many common antimicrobial agents such as the β-lactams [1]. In cattle, *M. bovis* is a causative agent of pneumonia, arthritis, otitis media, and reproductive disease and is a contributor to the bovine respiratory disease (BRD) complex, also known as ‘shipping fever’, which is a major source of morbidity, mortality and financial loss in calf and feedlot operations. Additionally, *M. bovis* is capable of persisting for the life of a colonized animal, which may remain asymptomatic while acting as a source of infection for herdmates or offspring [1,2].

Of note, many of the antibiotics to which *M. bovis* shows resistance are not licensed for usage in treating *M. bovis* infections [3] but may be used in the treatment of other bovine bacterial pathogens. Given the asymptomatic nature of many *M. bovis* infections, and the high rates of colonization when animals are co-mingled (potentially over 90%) [4,5], conditions are favourable for the development of multi-drug resistant strains. With global rates of antimicrobial resistance increasing, understanding the molecular mechanisms underlying antimicrobial resistance, particularly for multi-drug resistant (MDR) strains, is critical for determining effective treatment [6], or potentially to design treatment protocols that use evolutionary approaches to counter or reverse antimicrobial resistance [7].

Unlike other members of the BRD complex such as *Mannheimia haemolytica*, *Histophilus somni* or *Pasteurella multocida*, *M. bovis* is not known to possess defined antimicrobial resistance genes [8] but appears to have the molecular mechanisms of its resistance rooted in point mutations within several ribosomal and topoisomerase genes. Previous studies have used whole-genome sequencing (WGS) paired with minimum inhibitory concentration (MIC) testing to establish that mutations within *gyrA* and *parC* are linked to increased resistance to fluoroquinolones, that increased resistance to spectinomycin and the tetracyclines is linked to *rrs1-rrs2* (16S rRNA gene) mutations, and that *rrl1-rrl2* (23S rRNA gene) mutations are linked to increased resistance to florfenicol, lincosamides, macrolides and pleuromutilins [9], with *rrl3* (23S rRNA gene) also implicated in macrolide resistance [10].

In a large-scale MIC study of *M. bovis* strains isolated between 1978 to 2009, fluctuations in antimicrobial susceptibility over time were observed [3] with the MIC50 values, and thus resistance, increasing for several tetracycline and macrolide-class antimicrobial drugs over the span of the study. Additionally, associations between MIC50 values were observed for different antimicrobials; although sequencing these isolates fell beyond the scope of the study, the association between lincosamides, pleuromutilins and florfenicol in terms of *rrl1-rrl2* mutations was mirrored by a similar observed association in MIC50 values with these historical samples.

A high MIC *M. bovis* was isolated from lung tissues of a two-week old male Holstein calf submitted to the Animal Health Lab in July of 2019 for post-mortem examination. In the interest of determining possible genetic factors for this high level of resistance, whole-genome sequencing using the Illumina MiSeq platform was conducted, in tandem with WGS of a historical isolate of *M. bovis* (1982-M6152) previously categorized as low MIC for most antimicrobials [3]. The MICs and sequence data for both isolates were compared to *M. bovis* strain PG45, a reference strain with a fully sequenced and circularized genome, in order to identify any gaps in sequencing coverage, to determine if any new genes were present in the isolates as opposed to a reference strain, and to better elucidate which mutations in the high MIC isolate were potentially significant for AMR by ruling out any shared mutations with the low MIC isolate.

## 2. Results

### 2.1. MIC Testing

Cultures of two isolates of *M. bovis* (1982-M6152 and 2019-043682) and *M. bovis* strain PG45 (used as a reference strain) were tested in triplicate for minimum inhibitory concentration of 16 antimicrobials (Table 1), with the results of MIC testing identical within each triplicate. Relative to *M. bovis* PG45, isolate 1982-M6152 shows a two-fold increase in MIC for oxytetracycline but is otherwise identical in response to other antimicrobial compounds. Isolate 2019-043682 shows increased MICs for multiple fluoroquinolones, macrolides and tetracyclines, as well as a lincosamide, a pleuromutilin and two inhibitors of protein synthesis (gentamicin and florfenicol). For aminoglycosides the results are mixed, with increased MIC observed for spectinomycin, but no change in MIC for neomycin. All three strains have high MICs for sulfonamides, although they retain a low MIC for combination trimethoprim/sulfa.

### 2.2. Whole-Genome Sequencing

Raw sequencing yield for the two sequenced isolates was 167.8 Mb for 1982-M6152 (GenBank accession: CP058969), and 54.02 Mb for 2019-043682 (GenBank accession: CP058968). Given the sequencing yields and the documented genome size of 1.003 Mb for *M. bovis* PG45 (GenBank accession NC_014760.1), raw sequencing coverage (where C = yield/genome size) was 167× for 1982-M6152 and 54× for 2019-043682, which is sufficient for analysis of mutations and SNPs. Assembled using SPAdes 3.9.0 [11] in Illumina’s BaseSpace hub (Illumina, Inc., San Diego, CA, USA) genome sizes were 978,895 bp for 1982-M6152 and 941,076 bp for 2019-043682. Also within BaseSpace, core genome multilocus squence typing (cgMLST) was conducted for both isolates sequenced, using Bacterial Analysis Pipeline v1.0.4 [12]. Isolate 2019-043682 had an undescribed cgMLST type (ST10-like) while isolate 1982-M6152 had the same cgMLST type (ST17) as PG45.

**Figure 1:**
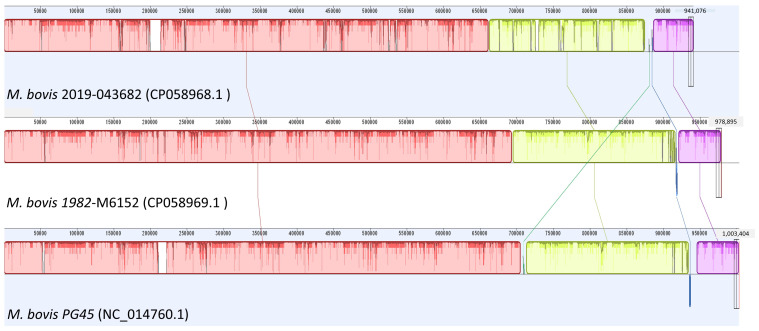
Graphical output of multiple sequence alignment (Mauve, Geneious 11) for *M. bovis* isolates 2019-43682, 1982-M6152 and *M. bvis* strain *PG45* with GenBank accessions displaying depth of sequencing and areas with large deletions.

Assembly and multiple sequence alignment (MSA) of both isolates with *M. bovis* PG45 in Geneious 11(Biomatters, Auckland, New Zealand) (Figure 1) revealed that 2019-043682 had 91% sequence similarity to PG45. 1982-M6152 had 97.2% sequence similarity to PG4.

Annotation of the MSA in MegAlign using feature data for PG45 identified 878 features (MegAlign’s term: CDS, in more general usage) in strain PG45, with divergences by isolate summarized in Table 2. Features were reported as Identical by MegAlign if they were 100% identical to PG45, with 100% coverage. Features were reported as Not_Mapped by MegAlign if their % identity score fell below 95%. Unmapped features have been further categorized by the researchers as excised, truncated or highly variable based on their % coverage score (Table 2). Isolate 1982-M6152 had 696 features identical to PG45, 105 with substitutions, 37 with insertions or deletions and 39 reported as Not_Mapped. Of these 39, 24 were excised, 11 truncated and 4 present but highly variable. Isolate 2019-043682 had 183 identical features, 471 with substitutions, 50 with insertions or deletions, and 173 reported as Not_Mapped. Of these, 81 were excised, 48 were truncated, and 44 were present but highly variable. Although several features were excised in the isolates relative to PG45, no unique features were identified in either isolate that were absent from PG45.

To determine which mutations could potentially alter gene function, further analysis of the two isolates using DNAStar’s ArrayStar software revealed 3285 individual nonsynonymous mutations relative to *M. bovis* PG45 in total between the two isolates, across 513 genes and pseudogenes. Isolate 1982-M6152 contained 81 genes with non-synonymous mutations, 20 of which were disrupted. Isolate 2019-043682 contained 507 genes with non-synonymous mutations, with 67 genes disrupted. 17 genes with non-synonymous mutations were common to both isolate 1982-M6152 and isolate 2019-043682, with 14 of the mutations identical between the 2 isolates. Four genes with disrupting mutations were common to both isolates, with an insertion mutation in gene MBOVPG45_RS03940 (insertion TTGT, PG45 genome reference position 918372) identical between isolates. A subset of 55 genes (Table 3) containing NSMs was selected for further consideration based on the functional role of the genes and known mechanisms of antimicrobials, through consultation of the Comprehensive Antibiotic Resistance Database (CARD) and literature review [13]. Additionally, 22 genes were identified as ABC transporter system genes (Table 4) although the lack of available characterization has led to them being grouped separately for discussion. A full list of nonsynonymous mutations, their sequence and their positions is available as supplementary data (Supplementary Tables S1 and S2), as well as an expanded version of Table 3 (Supplementary Table S3) containing gene descriptions.

## 3. Discussion

The recent isolate 2019-043682 had significantly elevated MICs for multiple fluoroquinolones, macrolides and tetracyclines, as well as a lincosamide, a pleuromutilin, spectinomycin, and two inhibitors of protein synthesis (gentamicin and florfenicol), indicating multi-drug resistant *M. bovis* can emerge in the field.

Of the *M. bovis* genes previously linked by Sulyok et al. with AMR for various classes of antimicrobial, two sites linked with fluoroquinolone resistance (*gyrA* and *gyrB*) display multiple non-synonymous mutations (NSMs) for the high-MIC isolate 2019-043682 and no NSMs for the low-MIC isolate 1982-M6152. *ParC*, likewise associated with fluoroquinolone resistance, shows 18 unique NSMs in the isolate 2019-043682, and a single NSM in 1982-M6152, which is shared with the 2019-043682, therefore the shared single NSM is unlikely to be contributory to the elevated MICs. Although genes *rrs1-rrs2* and *rrl1-rrl2* were associated with AMR for tetracyclines, spectinomycin, macrolides, lincosamides and pleuromutilins, there are no NSMs for them in the isolate 2019-043682 despite the elevated MIC values, suggesting additional genetic events may be associated with AMR for these antimicrobials.

For antimicrobials where an observed increase in MIC was not matched with a previously identified *M*. *bovis* resistance-associated mutation, genes identified as AMR-associated in other species, as well as genes within the same functional groups are likely candidates for AMR association. Beyond the genes previously associated with AMR in *M. bovis*, an additional 510 features contain non-synonymous mutations. Assigning these genes to functional groups allowed us to exclude pseudogenes and genes coding for uncharacterized and hypothetical proteins. Also excluded were genes whose NSMs were identical between isolates 2019-043682 (high MIC) and 1982-M6152 (low MIC). Of the 149 genes remaining, we focused on a subset of 55 genes with nonsynonymous mutations within functional roles known to be involved in antimicrobial resistance [13]: protein synthesis and topoisomerases. Additionally, 22 genes with NSMs were identified as ATP binding cassette (ABC) transporter system genes. These genes, although lacking full characterization, are nonetheless included in the discussion as targets for future analysis, due to the role of efflux pumps, particularly ABC transporters, in AMR.

### 3.1. Protein Synthesis

Interference with protein synthesis is a primary method of action for the antimicrobials, with different antimicrobials interfering at different stages of synthesis, and at different locations within the ribosome complex.

#### 3.1.1. Methyltransferases

RNA methyltransferases methylate specific bases within ribosomal RNA, altering the physical structure of binding sites and other active sites within the ribosomal subunits [16,29,30]. Mutations within the 16S RNA methyltransferase family are known to confer aminoglycoside resistance within other bacterial species [16], and five genes (*rsmA, rsmD, rsmH, rsmI* and MBOVPG45_RS02280, a 16S uracil methyltransferase) within isolate 2019-043682 contain NSMs not found in isolate 1982-M6152. The 23S methyltransferase *rlmA* has been associated with AMR for tylosin [29], and while *rlmA* is wildtype in isolate 2019-043682, the related 23S methyltransferases *rlmB, rlmD* and *rlmH* contain 5, 11 and 1 unique NSMs respectively. *RlmB* has also been identified as a potential site for AMR mutations based on an analysis of its structure [15], although no mutational studies have been conducted. For tRNA methyltransferases, *trmD* is implicated in multi-drug resistance [31]: it is wildtype in isolate 2019-043682, but two related tRNA methyltransferases (*trmB* and MBOVPG45_RS00465, a cytidine methyltransferase) contain NSMs, with *trmB* containing a gene disruption.

#### 3.1.2. Ribosomal Proteins

Mutations in *rpsC* and *rpsJ,* components of the 30S ribosomal subunit, are known to confer tetracycline resistance [18,20]. *RpsC* contains a single NSM in isolate 2019-043682 that is unique from the two NSMs observed in isolate 1982-M6152, but *rpsJ* is wildtype in isolate 2019-043682 and contains a single NSM in isolate 1982-M6152 that is therefore unlikely to influence MIC values. *RpsB* and *rpsE* contain 3 and 2 NSMs in the 2019 isolate, with a single, separate NSM present for *rpsE* in the 1982 isolate: mutations in these genes have been linked with aminoglycoside resistance [17,19], Additionally, five other 30S ribosomal proteins (*rpsD, rpsG, rpsP, rpsS*, and *rbfA*) contain NSMs in isolate 2019-043682.

Of the 50S ribosomal proteins with observed NSMs, *rplD* and *rplV* have been previously associated with macrolide resistance in *Clostridium perfringens* and two *Campylobacter* species [22,23] with *rplD* containing ten separate NSMs in the isolate 2019-043682. The 50S subunit gene *rplC*, where mutation has been previously associated with pleuromutilin resistance [32], contains a single NSM in the 2019 isolate, unique from the two NSMs found in the 1982 isolate. 50S ribosomal proteins mutations have also been linked with resistance to lincosamides, macrolides and phenicols [32]: an additional five genes (*rplB, rpmE*, MBOVPG45_RS00445, MBOVPG45_RS03525 and MBOVPG45_RS01360) coding for 50S ribosomal proteins contain NSMs in isolate 2019-043682 while remaining wildtype in isolate 1982-M6152. Among these, *rpmE* has been linked with multi-drug resistance [24].

#### 3.1.3. Aminoacyl-tRNA Synthetases

While none of the antimicrobials used in MIC testing in this study target them, aminoacyl-tRNA synthetases, also known as tRNA-ligases, are enzymes which attach individual amino acids to their corresponding tRNAs and are a target of interest for antimicrobial development [33]. Isolate 2019-043682 contains 22 tRNA-ligase genes with NSMs, of which ileS (a known target for pseudomonic acid) [27] is disrupted, as is alaS (a novobiocin target) [25], in addition to a glutamate-tRNA ligase (MBOVPG45_RSO1150) and a methionine-tRNA ligase (MBOVPG45_RS03150). *AsnS* and *pheS* have been linked with multi-drug resistance [26] and contain one and two unique NSMs in isolate 2019-043682, respectively. Three of these genes (alaS, ileS and MBOVPG45_RS02170, a threonine-tRNA ligase) contain different NSMs in isolate 1982-M6152, illustrating that the presence of an NSM on its own is not sufficient for AMR, and deeper investigation into changes in protein structure and function are required. LysS, containing 3 NSMs in isolate 2019-043682, has been identified as a gene contributing to methicillin resistance in MRSA [31]: as a β-lactam, methicillin is not used in the treatment of mycoplasma infections, but co-infection with *M. bovis* containing a potential AMR-associated mutation raises the possibility of horizontal gene transfer to a normally susceptible species.

### 3.2. Topoisomerases

In addition to *gyrA, gyrB* and *parC* discussed by Sulyok et al. (2017), *parE* mutations are also involved in fluoroquinolone resistance [14] and the isolate 2019-043682 contains four NSMs within the *parE* gene. While *topA*, a type I DNA topoisomerase, has not been linked with AMR previously, the gene, which is wildtype in isolate 1982-M6152, contains a single nucleotide “A” insertion at nt 1762 of the *topA* gene in isolate 2019-043682, which results in a *topA* (612–614 VK *) to *topA* (612–613 S *) mutation, likely a gene disrupting mutation. As bacterial topoisomerase I is a target of interest for antimicrobial development [34,35,36], screening for mutations affecting *topA* may be of future value to researchers and clinicians.

### 3.3. Bacterial Efflux Pumps: ABC Transporters

Bacterial efflux pumps are a class of membrane transport proteins whose role is the removal of toxic substances or metabolites from within the bacterial cell: It is estimated that 5–10% of all bacterial genes are involved in transport, with efflux pumps specifically comprising a large proportion of these transporters [37]. Of the two classes of efflux pump, primary and secondary, the primary transporters use ATP hydrolysis as an energy source, and are also known as ATP binding cassette transporters, or ABC transporters [38]. They are more commonly implicated in resistance to a single drug or category of drugs, although instances of multi-drug resistant ABC transporters have been described 200 [16,38].

As summarized in Table 4, 22 ABC transporter genes contain NSMs in isolate 2019-043682, one of which (MBOVPG45_RS03705) contains a gene-disrupting mutation. Three (MBOVPG45_RS01775, MBOVPG45_RS02905 and MBOVPG45_RS04315) also contain NSMs in isolate 1982-M6152. Although none are previously identified as SDR- or MDR- involved in *M. bovis*, the wide range of antimicrobials affected by efflux pumps suggests that this may be an area of interest for future research. While 8 non-ABC membrane transport proteins with NSMs were identified in isolate 2019-043682 (Supplementary Table S2), none has been characterized sufficiently to determine their potential as secondary efflux pumps and have thus been excluded from discussion.

### 3.4. Future Directions

Within the 55 genes selected for additional study based on functional role and the 22 ABC transporter genes, the 40 genes identified by their organism (eg., MBOVPG45_RS00380) rather than a common name limit the utility of a literature review or database search for assessing AMR potential. Although beyond the scope of the current study, a BLAST search for each gene to identify homology with other organisms could permit more detailed characterization of these genes and thus allow for a more thorough search of existing research into AMR. This would be of particular value for the ABC transporters, as all 22 identified as potential AMR associations due to their mutations are given *M. bovis*-specific identifiers. MBOVPG45_RS04315, with 89 separate NSMs in the high-MIC isolate, is a particularly strong candidate for a homology search.

As an initial foray by the research group into whole genome sequencing, the sequencing of a pair of high and low MIC isolates and the use of a fully-characterized reference strain (PG45) as a scaffold for assembly and annotation allowed us to determine which genes and which NSMs were non-contributory to the high MIC observed in the 2019 isolate, and allowed us to determine that no additional genes were present relative to the reference strain. Sequencing additional high-MIC strains of *M. bovis* as they arise in the future will allow us to develop further evidence in support of AMR association for the subset of genes identified and may uncover additional candidate genes for AMR association. Likewise, selecting historical strains for WGS that are high or low MIC for specific antimicrobials may allow for further refinement or expansion of the list of AMR-association candidates.

## 4. Materials and Methods

As a non-interventionary study, prior approval from the University of Guelph Research Ethics Board was not required for this research.

### 4.1. Culture & Isolation of Mycoplasmas

The body of a two-week old male Holstein calf was submitted to the Animal Health Lab in July of 2019 for post-mortem examination. Histologically, no lesions indicative of mycoplasma pneumonia were observed within the lungs. Culture and isolation of *M. bovis* AHL# 2019-043682 from the calf lung tissue was conducted as follows: The lung tissue submitted was perforated repeatedly using a sterile dry swab to collect sample material for broth (pig serum, horse serum and ureaplasma broths) and agar plate (pig serum agar, yeastolate agar, ureaplasma agar) culture [39]. Mycoplasma agar plates were incubated at 37 °C with 5–7% CO2 and 80–100% relative humidity. Ureaplasma agar plates ware incubated at 37 °C anaerobically. All broth cultures were incubated aerobically at 37 °C. Plates were read at 48–72 h intervals using a transilluminated stereomicroscope. Broth tubes were visually inspected for growth and pH change at 18–24 h intervals, and were subcultured twice, at 48–72 h growth and at 48–72 h following the first subculture. Agar plates were subcultured if suspicious growth was observed during reading. Following isolation, species identity as *M bovis* was confirmed using goat anti-rabbit/fluorescein isothiocyanate (GAR/FITC)-labelled antiserum fluorescent antibody staining [39]. Blocks of agar containing pure isolate were cut and stored at −80 °C for long term storage. Isolated 1982-M6152, an isolate of *M. bovis* from 1982 stored at −80 °C and identified in a previous study [3] as low MIC for most antimicrobials, was propagated and tested by WGS and MIC retesting.

### 4.2. MIC Testing

Minimum inhibitory concentration (MIC) testing was conducted on *M. bovis* isolates 2019-043682, 1982-M6152 and strain PG45 in triplicates for each isolate using previously described procedures [3], and using *M. bovis* isolate 227, an internal laboratory reference strain, as a control. Briefly: each isolate was first inoculated into 4 mL Mycoplasma MIC broth and incubated 48–72 h at 37 °C aerobically, before being frozen at −80 °C.

Following this incubation period, a colour-changing unit (CCU) and colony forming unit (CFU) count were setup to determine the number of CCU’s in the frozen aliquots. A 10-fold serial dilution was prepared for each isolate using Mycoplasma MIC broth, with 200 µL total volume in each of 12 wells. 10µL of the first 6 dilutions were plated onto Hayflick’s agar, and both the serial dilutions and agar plates were incubated for 48–72 h at 37 °C with 5–7% CO_2_ and 80–100% relative humidity. Both serial dilutions (lowest serial dilution showing a blue-red colour change) and agar plates (colonies counted using a stereomicroscope) were read after 48–72 h, and the CCU and CFU counts of the isolates were calculated accordingly.

A frozen aliquot was thawed and diluted in tubes of Mycoplasma MIC broth in successively larger volumes so that at least 25 mL of a 103–105 CCU/mL dilution was achieved. MIC testing was setup by inoculating 200 µL of this dilution into every well of a Sensititre BOPO6F microtitre plate. The Sensititre plate was incubated at 37 °C with 5–7% CO_2_ and 80–100% relative humidity for 24–72 h, until the positive control wells showed a blue-red colour change. At this point the Sensititre plate was read, and any wells showing a blue-red colour change were noted. The MIC for each antibiotic was calculated as the lowest concentration of drug that suppressed growth. After the Sensititre plate had been inoculated, the CCU and CFU counts of the inoculum were determined as previously described.

### 4.3. Nucleic Acid Extraction

For the isolates 1982-M6152, 2019-043682 and *M. bovis* PG45, 100 µL of a broth culture was extracted on the Applied Biosystems MagMAX 96 automated nucleic acid extraction platform (Applied Biosystems, Foster City, CA, USA) using the Low Cell Content protocol for the MagMAX Pathogen DNA/RNA kit (Applied Biosystems). Samples were eluted in a final volume of 90 µL, using the elution buffer provided with the kit, then held at −20 °C until prepared for WGS.

### 4.4. Whole Genome Sequencing & Bioinformatics

A 2 × 251 paired end sequencing reaction was conducted on the Illumina MiSeq platform (Illumina, San Diego, CA, USA) using a Nextera XT kit (Illumina) and associated protocols for whole genome sequencing (Illumina Custom Protocol Selector, Illumina Inc.) Quality filtering and assembly of FASTQ files was done on instrument and then uploaded to Illumina’s BaseSpace storage and computing cloud. On BaseSpace, genome assembly was conducted using SPAdes Genome Assembler v3.9.0 [11], and MLST assignment of two isolates using the Bacterial Analysis Pipeline v1.0.4 [12].

The assembled FASTQ files were then downloaded from BaseSpace, the adapter for the Nextera XT Kit (CTGTCTCTTATACACATCT) trimmed, then the genomes were assembled using bioinformatics software DNAstar (V17) (DNASTAR, Madison, WI, USA) using NGS-Based Reference-guide (small genomes, contigs) of Hybrid reference-guide/de novo genome assembly function against and annotated with features from reference genome *M. bovis* PG45 (GenBank Accession: NC_014760.1). Multiple genome alignment was performed using Mauve Genome of Geneious v11 (Auckland, New Zealand) with automatically calculated seed weight and automatically calculated minimum LCB score. SNPs were analyzed with MegAlign, DNASTAR’s ArrayStar v14 (DNASTAR, Madison, WI, USA), and Geneious v11 to identify deleted and truncated genes, SNPs and non-synonymous mutations relative to *M. bovis* PG45. Gene features were then tabulated in a spreadsheet (Supplementary Table S2) and further annotated by searching NCBI’s Gene database to identify their functional roles where possible. Named genes with functional roles in protein synthesis and topoisomerase structure that contained NSMs or disruptions in the 2019 isolate were selected for additional study, using the CARD database [13] and literature review (Google Scholar, keywords used: “antimicrobial resistance”, “antibiotic resistance”, AMR, and the gene name or functional group, I.e. “rplD” “antimicrobial resistance”) to identify mechanisms of antimicrobial resistance, and *M. bovis* gene homologues with AMR association in other organisms.

## 5. Conclusions

This study identified 55 genetic events of nonsynonymous mutations and gene disruptions linked to *M. bovis* AMR. Future studies are warranted to further analyze these candidate genes, identifying the effects of the altered amino acids on protein structure and their link to AMR. Additionally, mutated genes identified in this study but currently uncharacterized may be assigned to functional groups in future.

## Figures and Tables

**Table 1:** Average results of MIC testing for two isolates of Mycoplasma bovis, compared to reference strain PG45, by µg of antimicrobial compound required to inhibit growth. Isolates and the reference strain were tested in triplicate with identical results within each triplicate for all antimicrobials tested.

Antimicrobial	PG45	2019-043682	1982-M6152
Neomycin	>32	>32	>32
Spectinomycin	<8	16	<8
Trimethoprim/Sulfa	>2/38	>2/38	>2/38
Danofloxacin	0.5	>1	0.5
Enrofloxacin	0.5	2	0.5
Clindamycin	<0.25	>16	<0.25
Tilmicosin	<4	>64	<4
Tulathromycin	8	>64	8
Tylosin Tartrate	1	>32	1
Tiamulin	1	8	1
Gentamicin	8	16	8
Florfenicol	4	>8	4
Sulphadimethoxine	>256	>256	>256
Chlortetracycline	<0.5	>8	<0.5
Oxytetracycline	<0.5	>8	1
Ceftiofur	>8	>8	>8

**Table 2:** Summary of variation of isolates from *M. bovis* PG45, by feature count (CDS), generated using MegAlign. v17 for multiple sequence alignment.

Genetic Events		1982-M6152	2019-043682
Variation types	Identical	696	183
	Deletion	8	18
	Deleted_end_3prime	3	3
	Deleted_end_5prime	1	3
	Indel	3	4
	Insertion	22	22
	Not_Mapped	39	173
	Substitution	105	471
	**TOTAL**	877	877
Unmapped features	Excised (<5% coverage)	24	81
	Truncated (5–95% coverage)	11	48
	Highly variable (>95% coverage)	4	44
	**TOTAL**	39	173

**Table 3:** Count of non-synonymous mutations (NSMs) relative to *M. bovis* PG45, by gene and by isolate, for a subset of NSM-containing genes identified as potentially associated with antimicrobial resistance.

Functional Role:	Gene:	1982-M6152	2019-043682	Associated AMR:		Reference:
Topoisomerases	gyrA	0	8	fluoroquinolones		[9]
	gyrB	0	6	fluoroquinolones		[9]
	parC	1 *	19 *	fluoroquinolones		[9]
	parE	0	4	fluoroquinolones		[14]
	topA	0	1 ^			
**Protein Synthesis:**						
*Methyltransferases:*	MBOVPG45_RS00465		0	2		
	MBOVPG45_RS00470		0	7		
	MBOVPG45_RS02280		0	4		
	rlmB		0	5	Predicted AMR	[15]
	rlmD		0	11		
	rlmH		0	1		
	rsmA		0	6	aminoglycosides	[16]
	rsmD		0	1	aminoglycosides	[16]
	rsmH		0	5	aminoglycosides	[16]
	rsmI		0	5	aminoglycosides	[16]
	trmB		0	4 ^		
*30S Ribosomal Proteins*	rpsB		0	3	aminoglycosides	[17]
	rpsC		2	1	tetracyclines	[18]
	rpsD		0	2		
	rpsE		1	2	aminoglycosides	[19]
	rpsH		0	1		
	rpsJ		1	0	tetracyclines	[20]
	rpsP		0	2		
	rpsS		0	1		
	rbfA		0	1		
*50S Ribosomal Proteins*	MBOVPG45_RS00445		0	1		
	rplB		0	1		
	rplC		0	1	pleuromutilins	[21]
	rplD		0	10	linezolid	[22]
	MBOVPG45_RS03525		0	2		
	rplV		0	1	macrolides	[23]
	MBOVPG45_RS01360		0	1		
	rpmE		0	1	MDR	[24]
*tRNA ligases*	alaS		1	8 ^	novobiocin	[25]
	MBOVPG45_RS01640		0	5		
	asnS		0	1	multi-drug resistance	[26]
	MBOVPG45_RS00205		0	9		
	MBOVPG45_RS01150		0	6 ^		
	MBOVPG45_RS02730		0	2		
	MBOVPG45_RS02640		0	1		
	ileS		1	5 ^	pseudomonic acid	[27]
	MBOVPG45_RS03145		0	1		
	MBOVPG45_RS02255		0	16		
	lysS		0	3	methicillin	[28]
	MBOVPG45_RS03150		0	10		
	pheS		0	2	MDR	[26]
	MBOVPG45_RS00380		0	18		
	serS		0	1		
*tRNA ligases*	MBOVPG45_RS02170		1	8 ^		
	trpS		0	1		
	MBOVPG45_RS04210		0	9 ^		
	MBOVPG45_RS00740		0	7 ^		
	tilS		0	8		
	thiI		0	4		
	mnmA		0	5		

* Identical NSM; ^ Contains a gene-disrupting NSM.

**Table 4:** Count of non-synonymous mutations (NSMs) relative to *M. bovis* PG45, by gene and by isolate, for ABC transporter system genes potentially linked to the bacterial efflux pump mechanism of AMR. ^Gene contains a disrupting mutation.

Gene:	1982-M6152	2019-043682	Description:
MBOVPG45_RS00090	0	1	ABC transporter ATP-binding protein
MBOVPG45_RS00180	0	2	ABC transporter permease
MBOVPG45_RS00555	0	2	ABC transporter permease
MBOVPG45_RS00570	0	4	ATP-binding cassette domain-containing protein
MBOVPG45_RS00600	0	1	ATP-binding cassette domain-containing protein
MBOVPG45_RS01485	0	2	energy-coupling factor transporter transmembrane protein EcfT
MBOVPG45_RS01540	0	1	sugar ABC transporter permease
MBOVPG45_RS01545	0	4	ATP-binding cassette domain-containing protein
MBOVPG45_RS01720	0	1	ABC transporter permease subunit
MBOVPG45_RS01770	0	1	ABC transporter ATP-binding protein
MBOVPG45_RS01775	1	7	ABC transporter permease
MBOVPG45_RS02005	0	5	ABC transporter ATP-binding protein
MBOVPG45_RS02710	0	1	ABC transporter permease subunit
MBOVPG45_RS02715	0	2	ATP-binding cassette domain-containing protein
MBOVPG45_RS02905	1	1	ABC transporter permease subunit
MBOVPG45_RS03425	0	1	ATP-binding cassette domain-containing protein
MBOVPG45_RS03465	0	4	ABC transporter ATP-binding protein
MBOVPG45_RS03470	0	6	ABC transporter ATP-binding protein
MBOVPG45_RS03705	0	6 ^	carbohydrate ABC transporter permease
MBOVPG45_RS03710	0	1	sugar ABC transporter permease
MBOVPG45_RS04310	0	5	ABC transporter ATP-binding protein
MBOVPG45_RS04315	1	89	ABC transporter permease

^ Contains a gene-disrupting NSM.

## Supplementary Materials

The following are available online at https://www.mdpi.com/2076-0817/9/7/588/s1, Table S1: ArrayStar Output of Nonsynonymous Mutations, Table S2: Summary of NSMs, Table S3: AMR-Associated Gene Candidates. WGS sequence data for 1982-M6152 (Accession: CP058969) and 2019-043682 (Accession: CP058968) are available through GenBank.
